# Spontaneous gait phase synchronization of human to a wheeled mobile robot with replicating gait-induced upper body oscillating motion

**DOI:** 10.1038/s41598-022-20481-4

**Published:** 2022-09-29

**Authors:** Satoshi Yagi, Yoshihiro Nakata, Yutaka Nakamura, Hiroshi Ishiguro

**Affiliations:** 1 Graduate School of Engineering Science, Osaka University, 1-3 Machikaneyama, Toyonaka, Osaka 560-8531 Japan; 2 Present Address: Graduate School of Informatics, Kyoto University, Yoshida-honmachi, Sakyo-ku, Kyoto-shi, Kyoto 606-8501 Japan; 3 Present Address: Graduate School of Informatics and Engineering, The University of Electro-Communications, 1-5-1 Chofugaoka, Chofu, Tokyo 182-8585 Japan; 4 Present Address: RIKEN Information R&D and Strategy Headquarters, 2-2-2 Hikaridai Seika-cho, Sorakugun, Kyoto 619-0288 Japan

**Keywords:** Human behaviour, Engineering

## Abstract

Synchronization between humans is often observed in our daily lives, for example in breathing, in hand clapping in crowds, and in walking. It has been reported that pedestrian gait synchronization maximizes walking flow efficiency. As increasingly more mobile robots are being developed for practical use, it is important to consider how robots may impact pedestrian flows. While there is research on synchronization phenomena between humans and robots, gait synchronization between humans and robots has yet to be studied, particularly synchronization occurring with wheeled humanoid robots while moving. In this paper, we investigated the gait phase synchronization between humans and a wheeled mobile humanoid robot, which moved its upper body in three distinct types of motion patterns: (1) no-motion, (2) arm-swinging (as is common for typical mobile humanoids), and (3) arms-swinging in addition to periodic vertical-oscillation similar to the human upper body movement while walking. Rayleigh test was performed on the distribution of the obtained gait phase differences under each condition and a significant distributional bias was confirmed when participants were walking with the robot that performed both arm-swinging and vertical-oscillation of the upper body. These results suggest that humans can spontaneously synchronize their gaits with wheeled robots that utilize upper body oscillating. These findings can be important for the design of robot-integrated urban transportation systems, such as train stations and airports, where both humans and robots are mobile and a highly efficient flow is required.

## Introduction

Although robots designed to be integrated into factories are widely used in an industrial setting, it has been challenging to place robots in human living environments. This issue has been actively researched^1,2^. Previous robotics research has contributed to navigation methods to predict surrounding people’s trajectories and avoid collisions^3–11^.

These technologies are one-directional solutions from the robot’s perspective on how to perceive human action and act accordingly. The other direction is how humans recognize the robot’s actions and intentions accurately. There has been research on methods for communicating the robot’s movement intentions^12–17^; however, the people around the working robot may disregard them and instead feel that the robot is an obstacle^18^. Therefore, the effort of people required to recognize the robot’s action and intention should be minimized, and ideally, the level of effort should be unconscious^19^.

It is known that humans walking in large crowds can walk collectively, as they unconsciously synchronize their gait with the surrounding pedestrians^20–22^. This pedestrian synchronization is induced by a various factors such as hand-holding^23^, vibrations transmitted via a bridge^24^, and ambient music^25^. Even a small cognitive load, such as listening to a story when walking, enhances a gait synchronization^26^. Previous studies on pedestrian gait synchronization have reported that gait phase synchronization is more likely to occur in high-density groups and that pedestrian synchronization also maximizes the walking flow efficiency^27,28^. Additionally, it has been reported that there is a psychological aspect to synchronized side-by-side walking, as it improves the people’s impression of each other^29^.

Various studies have reported the importance of motion synchronization between robots and humans^30–34^. In research on gait, there has been a study on a walking assist device that enables a pedestrian to synchronize their gait by listening to the footsteps of a CG avatar displayed on a screen^35^. Another synchronization method found in another study is by vibrating a treadmill device in the vertical direction for the participant to synchronize gait through the shaking^36^. However, it is still not clear whether humans can synchronize their gaits through walking motion expressed by an actual mobile robot. If humans can synchronize a gait phase with robots, we can imagine applications with smooth robot–human mobility in our daily lives. In particular, if both the bipedal robots and the more common wheeled robots can synchronize gait phases by utilizing upper body motions, robots can move by taking advantage of the human ability to walk skillfully in the crowd. In order to utilize the benefits of pedestrian synchronization in wheeled mobile robots, we developed a wheeled humanoid that can perform pedestrian-like moving motions, including upper body oscillating induced by a gait.

**Figure 1:**
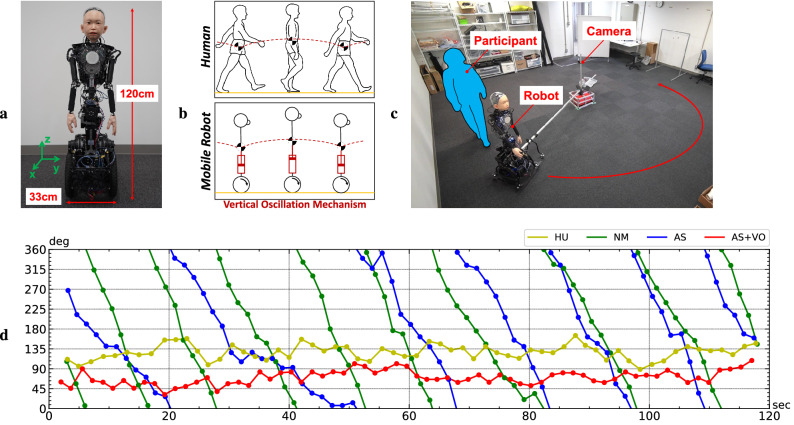
Overview of the experiment: (**a**) *ibuki*: a child mobile android robot with a human-like appearance. (**b**) a vertical-oscillation mechanism (VOM) of *ibuki*’s mobility unit which actuates its upper body vertically while moving, similar to the way the human upper body oscillates while walking. (**c**) The experimental setup: every participant walked behind a human (HU)/the robot with three types of upper body motion patterns (NM, AS, and AS+VO) (**d**) One of the representative obtained time-series phase difference graphs under four condition (Participant 3 in Fig. 3). The horizontal axis shows the measurement time (s) and the vertical axis shows the phase difference (deg). Yellow: HU, green: NM, blue: AS, and red: AS+VO condition.

In this paper, we investigated the synchronization of gait phases between a human and a wheeled humanoid robot. We implemented a periodic upper body motion in a wheeled child-like android robot (Fig. 1a), which oscillates its upper body vertically while moving (Fig. 1b), and measured the gait phase difference between the robot and a human walking behind the robot. In the experiment, participants walked in a circle behind the robot in a line (Fig. 1c), and their gaits were captured by a camera to obtain the phase difference between the two. For comparison, we conducted paired walking under four conditions: participants walking behind a human instead of the robot (“HU condition”), behind the robot with No-Motion (“NM condition”), behind the robot that its Arms-Swinging (“AS condition”), behind the robot that its Arms-Swinging adding Vertical-Oscillation of the upper body (“AS + VO condition”). After the experiment, we analyzed the bias in the phase difference distributions measured at each gait cycle. We then verified whether the gait phase synchronization occurred under each of the four conditions.

Before explaining the results, we must explain how we assess gait phase synchronization and the statistical analysis used in this paper. A human gait is periodic, with one walk cycle consists of one foot landing on the ground, swing in the air, and then landing on the ground again. Traditionally, one cycle of the gait phase $\phi$ (0–360 deg) is described as follows based on the right foot: heel-strike (0), middle-stance (90), pre-swing (180), middle-swing (270), and then back to the first heel-strike (360)^37^. As synchronization is a state in which two or more human gait cycles repeat oscillations while maintaining a certain angle, when two pedestrians’ gaits synchronize, the gait phase difference $\Delta \phi$ remains constant regardless of the walking time. This is called phase locking. Conversely, when they are not synchronized, their gait phases increase independently, and $\Delta \phi$ increases or decreases monotonically along the time. That causes the distribution to $\Delta \phi$ become uniformly distributed. Therefore, we analyzed that the measured gait phase differences $\Delta \phi$ were not uniformly distributed to validate whether the gait phases were synchronized. We used two analyses, Rayleigh test and Phase Locking Index (PLI) calculation. Rayleigh test is a method to test under which motion conditions synchronization occurs in the robot by testing whether the obtained circular data of $\Delta \phi$ is uniformly distributed. The PLI calculation is to further check the degree of synchronization under each participant in every condition.

## Results

Figure 1d shows one of the representative participants who spontaneously synchronized their gait phase with the human/robot under both the HU and AS+VO conditions. The horizontal axis shows the measurement time (s), and the vertical axis shows the gait phase difference (deg). No synchronization, no constant phase locking, was observed for NM (Fig. 1d, green) and AS (Fig. 1d, blue) conditions. The gait phase difference decreased monotonically in every gait cycle. Interestingly, for AS+VO (Fig. 1d, red) condition, we can observe synchronization between the human and the robot, similar to HU condition (Fig. 1d, yellow). The average gait phase difference was M = 127.6 ± SD 16.4 deg (Mean ± Standard Division) under HU condition and M = 70.2 ± SD 15.8 deg under AS+VO condition. As can be seen in Fig. 1d, when the gait phase synchronization occurs under a certain condition, the distribution of the gait phase difference is biased. In contrast, when the synchronization does not occur, the gait phase difference is uniformly distributed.

**Figure 2:**
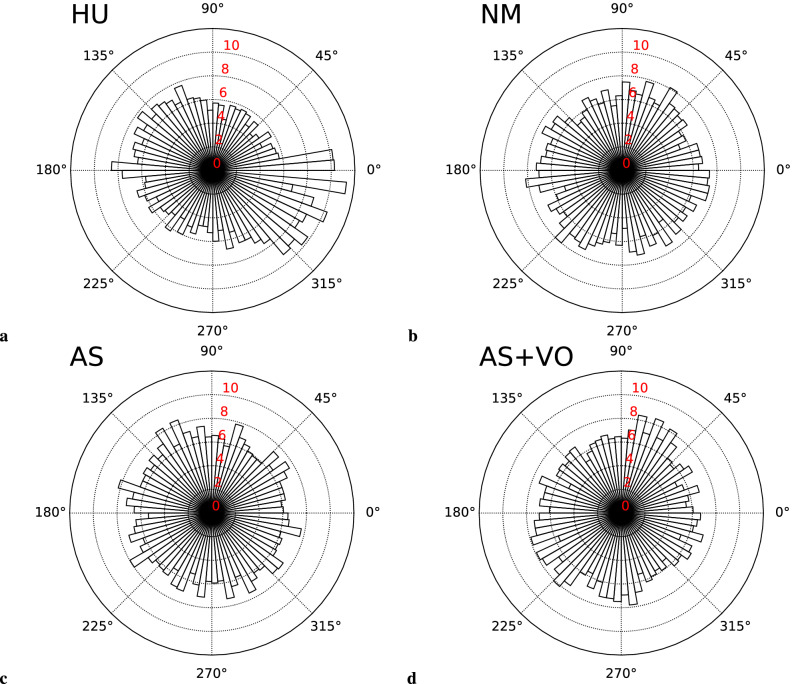
The circle histograms of four conditions which show the proportion of obtained gait phase differences at every five deg. The height in radius direction shows the normalized range of 0–12both HU (p < 0.0001) and AS + VO (p = 0.0485) conditions. At the same time, there was no significant distribution bias for NM (p = 0.9566) and AS (p = 0.0919) conditions.

Figure 2 shows the gait phase difference distributions of 1960, 1993, 2000, and 1989 gait cycles in total from 26 participants in HU, NM, AS, and AS+VO condition, respectively. The circle histogram shows the proportions of obtained gait phase differences at every 5 deg. The height in radius direction shows the normalized range of 0–12 condition (Fig. 2a), the distribution seems to be biased to the directions of 0 and 180 deg, indicating that the gait phases were synchronized in-phase and anti-phase. In the same way, in the AS + VO condition, the distribution seems to be biased to the upper-right and lower-left directions. To test the distributional bias of the gait phase differences, we performed Rayleigh test for each condition. We set the p-value to less than 0.05 to be statistical significance and confirmed that the distributional bias was significant (gait phases were synchronized) for both HU (*z* = 20.0663, $p<$ 0.0001) and AS+VO (*z* = 3.0261, *p* = 0.0485) conditions. At the same time, there was no significant distribution bias (gait phases were not synchronized) for NM (*z* = 0.0443, *p* = 0.9566) and AS (*z* = 2.3873, *p* = 0.0919) conditions.

**Figure 3:**
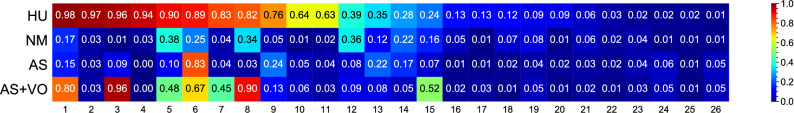
Heatmap of Phase Locking Index (PLI) for different participants (columns) in four conditions (rows). The number in a cell shows the PLI with the participant in each condition.

Figure 3 shows a heatmap of PLI. PLI is a dimensionless value which indicates the ratio the observed degree of synchronization to the maximum possible degree of synchronization. The PLI calculation was performed to confirm participants who were synchronized. The four conditions are indicated in rows from top to bottom, and the participants in columns from left to right (in order of higher PLI under HU condition). It can be seen that some participants in AS+VO condition gave a high PLI as well as HU condition. Especially, there were 11 whose PLI was higher than 0.5 in HU and 5 participants whose PLI was higher than 0.5 in the AS+VO condition.

## Discussion

In this paper, we investigated the synchronization of gait phases between humans and the wheeled humanoid robot. We implemented a periodic upper body motion in the wheeled child-like android robot and measured the gait phase difference between the robot and a human walking behind the robot under four conditions. We then analyzed the bias in the gait phase difference distribution under each condition by Rayleigh test. As a result, we confirmed the gait phase synchronization under the condition with human walking behind a robot with vertical-oscillation of the upper body. Interestingly, the gait phase synchronization was not observed in AS condition, in which the robot simply swing its arms, however, was observed in AS + VO condition, in which a simple vertical motion was added to the motion of AS.

**Figure 4:**
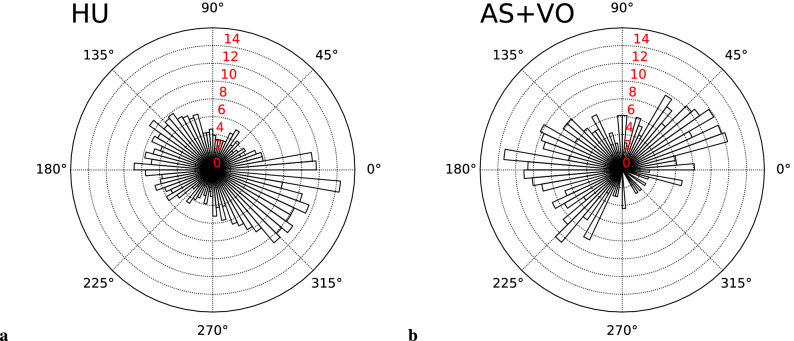
The circle histograms of the synchronized participant are only (PLI > 0.5) in HU and AS+VO conditions which show the proportion of obtained gait phase differences at every five deg. The height in radius direction shows the normalized range of 0–16% proportionally.

Although we observed the gait phase synchronization in AS+VO condition, the distribution in Fig. 2d is seen to be tilted to the upper right direction. Since we did not align the phases of the robot joints in the experiment, we confirmed the control delay which was about 0.2 s in the displacement of the VOM relative to the left shoulder joint. Considering that we set the robot’s natural angular frequency $\omega _0$ = 207 deg/s (see *f* in Table 1), there was a phase delay of 207 x 0.2 = 41.4 deg between the shoulder joint and the VOM. Instead of the left upper arm as the way of Fig. 2d, we redrew the distributions of the gait phase differences using the robot gait phase based on the VOM displacement at Fig. 4b. For a clear comparison, Fig. 4 shows the distributions of only participants whose PLI was higher than 0.5 under each condition. Both distributions have modes to the directions of 0 and 180 deg, indicating that the gait phases were synchronized in-phase or anti-phase. This result indicates that participants were synchronizing their gaits based more on the VOM than on the arm swing. This is consistent with the previous research which claimed the importance of the upper body swing during human interpersonal coordination^38–40^ and this is why the mode of the distribution shown by the synchronization in Fig. 2d is tilted to the upper-right (lower-left) direction.

**Figure 5:**
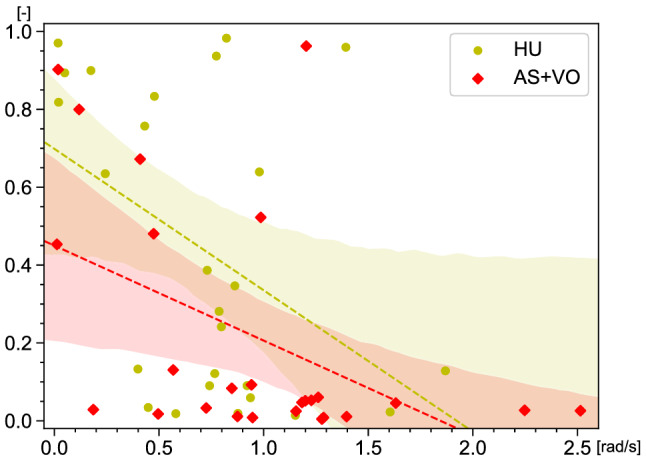
Scatter plot which shows the relationship between Phase Locking Index (PLI) [–] and the natural angular frequency difference $|\omega _{0,l} - \omega _{0,f}|$ [rad/s] under HU (yellow) and AS+VO (red) conditions. The dotted lines indicate the estimated PLI for $|\omega _{0,l} - \omega _{0,f}|$. The shaded regions represent 95% confidence intervals.

Next, we validate the factors of participants whose gait phases were synchronized in the HU and AS+VO conditions. Figure 5 shows the relationship between PLI and the natural angular frequency difference of the leading human/robot (leader) and each following participant (follower) $|\omega _{0,l} - \omega _{0,f}|$ under HU (yellow) and AS+VO (red) conditions. The average natural angular frequency of the human leader $\omega _{0,l}$ in the HU condition was M = 226.2 ± SD 13.3 deg/s and $\omega _{0,l}$ of the robot in the AS+VO condition was 207 deg/s. In addition, the average natural angular frequency of the follower $\omega _{0,f}$, which was calculated based on the data when a participant walked alone, was M = 261.9 ± SD 35.4. There were significant negative correlations between PLI and the natural angular frequency difference for both conditions (*r* = – 0.438, *p* = 0.0252 for HU, *r* = – 0.481, *p* = 0.0128 for AS+VO). Therefore, the participants who had a closer natural angular frequency to that of the human/robot leader, which means a smaller detuning^41^, synchronized their gaits more frequently. Considering that the difference between the angular frequency of the robot and participants was 54.9 on average, it is necessary to control the robot’s gait motion to fill this gap to synchronize the gait phase with more people. In the experiment, the robot moving ahead of participants did not obtain any information about the gait phases of the human who was following, however in practical use, it is important to synchronize the gait phases with more people by providing feedback on the gait cycles of people walking around the robot. Note that, the robot only needs to perceive and match the human gait cycle and the remaining adjustment of the gait phase can be left to the human side.

We would like to reiterate the importance of gait phase synchronization based on the experimental data obtained in this paper. At the result analysis, we observed gait interference of synchronized-participants at NM condition. While synchronized-participants walked in step with the robot in the AS + VO condition, in order to avoid colliding with the robot, their steps seemed to be disturbed. As a reference, we approximate the energy consumption ratio at NM to AS + VO condition for two group, non-synchronized and synchronized participants (PLI > 0.5 at AS + VO). As a result, the synchronized participants were consuming 1.51 times much more energy of walking under NM than that of AS+VO in average. The average for all participants was 1.23. See the Method section about the detail of the calculation $\eta$. Gait phase synchronization by expressing the robot’s human-like walking motion may be effective not only in improving the efficiency of the pedestrian flow, but also in reducing the walking energy consumed by the human side.

## Methods

### Participant

26 participants (Mean age 24.1 ± SD 7.9, 11 females and 15 males) were recruited for the experiment. All participants gave written informed consent. The protocol was approved by the ethics committee for research involving human subjects at the Graduate School of Engineering Science, Osaka University (#R1-6) and the methods were carried out in accordance with the approved guidelines.

### Robot

Figure 1a shows the child-like mobile android that we developed called *ibuki*^42^, which is comprised of two parts: a mobility unit (lower part) and the human-like upper body. *ibuki* is designed based on the dimensions of a 10-year-old Japanese boy and has a silicone skin-covered face and hands. The mobility unit, *ibuki* has a vertical-oscillation mechanism (VOM) which actuates its upper body vertically while moving (Fig. 1b)^43^. When a human moves two steps forward in one gait cycle, in each single support phase, the ankle and knee joints of each support leg flex and then extend, the human body oscillates twice on the sagittal plane in one gait cycle^44^. VOM mimics this upper body vertical oscillation induced by a human gait. This gait-like movement of the robot’s upper body driven by the VOM in the wheel-driven mobility unit is called “gait-induced upper body motion” in this paper.

### Motion generation

We extracted characteristic joint amplitudes and frequencies from a human gait to implement the robot’s gait-induced upper body motion. The author (176 cm) filmed himself walking on a treadmill at 0.83 m/s (3.0 km/h) treadmill speed for three minutes in 30 frames per second. At this speed, we confirmed beforehand that the robot was able to stably and safely move indoors with a human.

From the recorded video, joint position time series data was obtained by human pose recognition software MediaPipe^45^. After that, we calculated a time-series joint angle of the neck, the right shoulder and the right elbow in the pitch axis, the waist in the yaw axis, and a vertical displacement of the waist’s center position. It is known that human gait motions can be well described by the sum of sinusoidal functions using fast Fourier transformation^46^. Therefore, after all the data were filtered with a 3.3 Hz low-pass filter, we used fast Fourier transformation on these time-series data to acquire Fourier coefficients up the second terms for the amplitudes and frequencies. As the result, the robot’s joints $\varvec{\theta }(t)$ at time *t* were controlled by the following Eq. (1), where $\alpha$ was set as 0 or $\pi$ depending on the right or left body and the averages of the original joint angles $\varvec{{\bar{\theta }}}$ were added as the baselines.

1 $$\begin{aligned} \varvec{\theta }(t)=\varvec{A}\cos (2\pi t \varvec{f} + \varvec{\alpha })+\varvec{{\bar{\theta }}} \end{aligned}$$

Table 1 shows the calculated parameters for the gait-induced upper body motion. The left column shows the body joints which we controlled and each row shows parameters including amplitudes, frequencies, a phase difference, and a center angle of oscillation. The rotation directions of the pitch and yaw axes coincide with the rotations around the y and z-axes in Fig. 1a.

**Table 1:** The calculated parameters of each joint $\theta$, the amplitude *A*, frequency *f*, phase difference $\alpha$, and baseline ${\bar{\theta }}$ for the gait-induced upper body motion.

$\theta$	[Unit]	*A*	*f*	$\alpha$	${\bar{\theta }}$
Neck (pitch)	[deg]	0	–	–	$-28.3$
Shoulder (right, pitch)	[deg]	13.4	0.575	$\pi$	$-14.1$
Shoulder (left, pitch)	[deg]	13.4	0.575	0	$-14.1$
Elbow (right, pitch)	[deg]	5.47	0.575	$\pi$	$-28.6$
Elbow (left, pitch)	[deg]	5.47	0.575	0	$-28.6$
Waist (yaw)	[deg]	6.76	0.575	$\pi$	0
VOM	[mm]	18.7	1.15	$\tfrac{\pi }{2}$	0

For each condition, we controlled the following joints for the robot motions. At NM condition, we did not operate any joints. At AS condition, we operated both sides of the shoulders and elbow joints. At AS + VO condition, we operated the neck, waist, and VOM in addition to the shoulders and elbows. In all conditions, the wheels were driven at 0.83 m/s by a velocity control.

### Procedure

The experiment consisted of five walking sessions. In order, (1) the participant walked behind a human (HU condition), (2) the participant walked alone (this was not an experimental condition but for measuring a gait cycle when walking freely for Fig. 5), (3) the participant walked behind the robot with no motion (NM condition), (4) the participant walked behind the robot with the arms swinging (AS condition), (5) the participant walked behind the robot with the Arm-Swing adding Vertical-Oscillation of the upper body (AS + VO condition).

Each walking session (condition) lasted 2 min. Participants (as followers) walked the experiment room with following the human/robot (leader) ahead of them as shown in Fig. 1b. There was a fixed base in the experiment room with a rotatable lightweight beam at the center. The beam ensured that the distance between the center of rotation and the leader was kept 2.3 m for all conditions. Furthermore, it held the rotating camera in order to record the human gait. The camera was connected and controlled by a single board computer to capture photos of a human gait ($480 \times 680$ pixels, 30 fps) with time information during the experiment. We also recorded the robot’s joint reference angles and measured angles on 10 Hz in conditions with the robot.

In HU condition, participants walked behind a human (the author), leading and pushing the beam with the attached camera. In the free-walking session, participants walked alone and freely without any constraints, except for the author filming with the camera attached to the manually controlled beam. In NM, AS, and AS + VO conditions with the robot, participants walked behind the robot. In these sessions, the beam was connected to the robot’s mobile unit. The order of these three sessions was changed for every participant to ensure counterbalancing.

The human gait synchronization can be influenced by the surrounding sounds^25,47^. In order to eliminate the influence of the motor drive noise while the robot was moving, the participants wore noise-cancelling headphones and listened to white noise while walking. In addition, a participant’s gait was considered to be symmetrical for the analysis^48^.

### Analysis

One cycle of the gait phase (0–360 deg) is defined as follows: heel-strike (0 deg) in which the right heel touches the ground, middle-stance (around 90 deg) in which the right foot supports the upper body and the left foot swings forward, pre-swing (180 deg) in which the left heel touches the ground and the right foot leaves the ground, middle-swing (around 270 deg) in which the left foot supports the upper body and the right foot swings forward, and then back to the first heel-strike (360 deg) where the right heel touches the ground^37^.

For the analysis, we calculated the gait phase difference $\Delta \phi$ between the leader and follower for every gait cycle to determine if the two gaits were synchronized or not. In this paper, we defined the gait phase difference $\Delta \phi$ given by Equation 2. Here $t_{n, f}$ is the time when the follower’s gait phase reaches $\phi _{n, f}$ = 180 deg. Indexes *n*, *f* mean *n*-th gait cycle of the follower. $t_{n^\prime , l}$ is the time when the leader’s gait phase reaches $\phi _{n^\prime , l}$ = 180 deg in $n^\prime$-th gait cycle. Note that, $n^\prime$ was the closest gait cycle to *n*, which takes $|t_n - t_{n^\prime }|$ minimum.

2 $$\begin{aligned} \Delta \phi _n = {\left\{ \begin{array}{ll} 360 \frac{t_{{n, f}} - t_{{n^\prime , l}}}{t_{{n^\prime +1, l}}-t_{{n^\prime , l}}} &{} (t_{{n^\prime , l}} \ge t_{{n, f}})\\ \\ 360 \frac{t_{{n, f}} - t_{{n^\prime -1, l}}}{t_{{n^\prime , l}}-t_{{n^\prime -1, l}}} &{} (t_{{n^\prime , l}} < t_{{n, f}}) \end{array}\right. } \end{aligned}$$

To obtain $t_n$ of a human, the gait phase is calculated using the left leg angle from the captured human gait images. Firstly, two positions of a participant’s left waist and left knee were estimated by using image pose recognition (Mediapipe) in every frame to obtain the time-series with the left leg angle against the vertical direction. Next, a 3.3 Hz low-pass filter was applied to remove high-frequency noise from the time-series data. After that, we set $t_n$ the time when the angle value took minimum, which was a pre-swing phase $\phi _n$ = 180 deg of the *n*-th gait cycle.

To obtain $t_n$ of the robot the robot’s gait-induced upper body motion was generated based on a programmed gait phase which increases from 0 to 360 deg along with the real time. For NM condition, we simply used the time when *n*-th gait phase $\phi _n$ reached 180 deg on the program as $t_n$. On the other hand, for AS and AS + VO conditions, we had to consider that there was a small time delay between the moment when $\phi _n$ reaches 180 deg on the program and the actual time when the current flows to the motor and reaches the desired joint angle. Therefore, we decided to reference the sensor measurement of the robot’s left upper arm angle to obtain $t_n$. Specifically, we set $t_n$ the time when the left upper arm angle took minimum (pre-swing phase of the *n*-th gait cycle $\phi _n$ = 180 deg). For Fig. 4b, we calculated a time $t_n$ when the VOM displacement took a minimum value and we adjusted the gait phase difference by 207 deg/s $\times -0.2$ s = – 41.4 deg.

The natural angular frequency $\omega _0$ of a human was defined as $\omega _0 = \langle \omega _n \rangle$, here the angular frequency $\omega _n$ of *n*-th gait cycle was calculated as $\tfrac{360}{t_n-t_{n-1}}$. As we controlled the robot by the frequency *f* = 0.575 (see Table 1), we set the natural angular frequency $\omega _0$ as 360 × 0.575 = 207 deg/s for the robot.

To determine the degree of synchronization for each participant and condition, we calculated the Phase Locking Index (PLI)^49,50^. PLI is defined by Eq. (3) using *N* phase difference data. The value of PLI falls between 0 and 1, with 0 being completely unsynchronized and 1 being perfectly synchronized.

3 $$\begin{aligned} PLI = \frac{1}{N}\left|\sum _{k=1}^{N}e^{i\Delta \phi _k}\right| \end{aligned}$$

To approximate the energy consumption ratio $\eta$ during walking in NM to AS + VO condition by Eq. (4). *I* as the representative inertia of the body joints, the kinetic energy which the walking follower had was $\frac{1}{2}\ I\ {\omega _n}^2$. Here we assume all the body joints move at the same angular velocity and neglect the difference of the translational kinetic energy between two conditions. When we assume that followers consumed energy in the same way both when they increase or decrease the walking speed, the energy consumption from *n*-1-th gait cycle to *n*-th one was calculated as $\frac{1}{2}\ I\ |{\omega _n}^2 - {\omega _{n-1}}^2|$. The sum of the energy consumption obtained during the 120-s measurement in the experiment was taken as $\sum _n$.

4 $$\eta = \frac{{\sum\nolimits_{n} {\frac{1}{2}I\left| {\omega _{n} ^{2} - \omega _{{n - 1}} ^{2} } \right|_{{{\text{NM}}}} } }}{{\sum\nolimits_{n} {\frac{1}{2}I\left| {\omega _{n} ^{2} - \omega _{{n - 1}} ^{2} } \right|_{{{\text{AS + VO}}}} } }}$$
